# Refractory *Microascus* Bronchopulmonary Infection Treated with Olorofim, France

**DOI:** 10.3201/eid2911.230984

**Published:** 2023-11

**Authors:** Emmanuel Faure, Olivier Brugière, Sylvie Colin de Verdiere, Fanny Vuotto, Lucie Limousin, Emilie Cardot, Camille Cordier, Pauline Coulon, Dea Garcia-Hermoso, Olivier Lortholary, Fanny Lanternier

**Affiliations:** Université de Lille, Lille, France (E. Faure);; Centre Hospitalier Régional Universitaire Lille, Lille (E. Faure, F. Vuotto, C. Cordier, P. Coulon);; Hôpital Foch, Suresnes, France (O. Brugière, S. Colin de Verdière, L. Limousin, E. Cardot);; Institut Pasteur, Paris, France (D. Garcia-Hermoso, O. Lortholary, F. Lanternier);; University Hospital Necker for Sick Children, Assistance Publique-Hôpitaux de Paris, Paris (O. Lortholary, F. Lanternier)

**Keywords:** Microascus, olorofim, Microascus melanosporus, M. cirrosus pneumonia, respiratory infections, fungi, France

## Abstract

We report 3 cases of successful treatment of *Microascus* spp. bronchopulmonary infection in a multiple-traumatized patient and 2 lung transplant recipients in France. We emphasize the promising use of olorofim antifungal therapy in a rising context of intrinsically less-susceptible respiratory infections caused by mold.

The family Microascaceae includes genera *Microascus* and *Scopulariopsis*, opportunistic fungi that have caused respiratory infection associated with poor outcome and an attributable mortality rate of 85%–100% (*1*,*2*). Treatment of invasive *Microascus* infection is challenging because of its high resistance to available therapies. Olorofim, a reversible inhibitor of the enzyme dihyroorotate dehydrogenase, has shown in vitro activity against a variety of mold species, including azole-resistant *Aspergillus* (*3*,*4*) and *Microascus* spp. (*5*). We describe 3 cases of invasive *Microascus* respiratory infection in France that were treated with olorofim (Table). All patients gave informed consent for publication.

**Table:** Medical history and keypoints of 3 case-patients with refractory microascus bronchopulmonary infection, France*

Characteristic	Case 1	Case 2	Case 3
Age, y	17	61	65
Immunocompromised status	No	Lung transplant	Lung transplant
Years since transplantation	NA	4	6
Chronic lung allograft dysfunction	NA	Y (for 2 y)	Y (for 5 y)
Intensification of immunosuppressive drug regimen in medical history	NA	Antithymocyte globulin, steroids, rituximab, alemtuzumab, extracorporeal photophoresis	Steroids, rituximab, bortezomib
Maintenance therapy on the onset of *Microascus* infection	NA	Tacrolimus(C_0_ 4-6 ng/mL), everolimus (C_0_ 4-6 ng/mL), prednisone (5 mg/d)	Tacrolimus (C_0_ 4-6 ng/mL), Everolimus (C_0_ 4-6 ng/mL), prednisone( 5 mg/d)
Recent antifungal exposition <3 mo	None	Isavuconazole	Isavuconazole
Tolerance			
Clinical	No SSE	NA	No SSE
Biologic	No ELE	Drug interaction with tacrolimus and everolimus	No ELE

*ELE, elevated liver enzyme; NA, not applicable; SSE, significant side effect.

Case 1 occurred in a 17-year-old boy with unremarkable medical history who was found unconscious with inhalation pneumonia, bilateral hemopneumothorax, and bilateral thoracic drainage after falling from the top of a rice silo (Appendix Figure 1). On day 2, the patient underwent venovenous extracorporeal membrane oxygenation. On day 38, after 5 weeks of adapted antimicrobial treatment, thoracic computed tomography (CT) scan showed worsening of bilateral necrotizing pneumonia with abscess. Bronchoalveolar lavage (BAL) and several bronchial aspirations grew a restricted light-gray fungal colony (Appendix Figure 2), identified through the Paris National Reference Center as compatible with *Microascus melanosporus*; we initiated a combination of olorofim (180 mg 2×/d on day 1, followed by 90 mg 2×/d) and terbinafine (500 mg 2×/d) for 6 weeks (Appendix Table). Radiologic findings and general clinical status improved; we discontinued oxygen support after 2 weeks (day 73). The last CT scan showed complete healing of lung lesions (day 120). The patient was still alive 1 year later.

Case 2 occurred in a 61-year-old lung transplant recipient who sought care for respiratory deterioration and decline in respiratory function. He had recently received isavuconazole for bronchial colonization with *Aspergillus flavus*. Thoracic CT scan at admission showed a new alveolar consolidation in the left upper lobe (Appendix Figure 3); fibroscopy showed a recent-onset yellowish irregular lesion in the culminal bronchus (Figure, panel A). We isolated *M. cirrosus* from a culture of bronchial aspirate and BAL. We found no other disseminated lesions and retained the diagnosis of invasive pulmonary *M. cirrosus* infection. We initiated olorofim (90 mg 2×/d). We observed, as previously described (*6*), a moderate increase of both tacrolimus and everolimus blood through levels, which may have been caused by olorofim, a weak inhibitor of CYP 3A4. After 3 months of treatment, lung function slightly improved, CT scan showed a near-complete disappearance of the consolidation, and BAL culture was sterile. After 8 months of olorofim treatment, the endobronchial lesion was gone (Figure, panel B). *M. cirrosus* was found in BAL after 6 months of olorofim, but no more was cultured from BAL 7 months after treatment initiation. The patient was still being treated with olorofim at 9 months.

**Figure:**
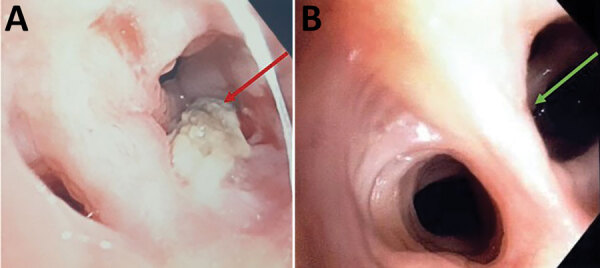
Macroscopic observation of endobronchial *Microascus cirrosus* lesion in patient in France with refractory microascus bronchopulmonary infection before (A) and after (B) olorofim treatment. Arrows indicate the lesion.

Case 3 occurred in a 65-year-old lung transplant recipient who sought care for dyspnea. He had experienced progressive decline of respiratory function and had a diagnosis of grade 3 bronchiolitis obliterative syndrome (BOS) linked to obstructive respiratory failure 6 years after transplant. He required permanent oxygen support. At admission, he received isavuconazole that continued for 3 months because of bronchial colonization with *A. fumigatus*. Thoracic CT scan results showed an unchanged pattern of BOS. Nevertheless, bronchial fibroscopy showed a new-onset bronchial lesion, necrotic and blackish in appearance, obstructing the origin of the culminal bronchus (Appendix Figure 4). We isolated *M. cirrosus* samples. Patient received a combination of oral terbinafine (500 mg 2×/d) and olorofim (180 mg 2×/d on day 1 followed by 90 mg 2×/d). After 3 months of treatment, bronchial fibroscopy showed an improvement of the bronchial lesion, and *M. cirrosus* was not found in respiratory specimens. The patient died from respiratory failure attributed to progression of BOS.

Use of olorofim for invasive *Microascus* spp. respiratory infection has not previously been reported with a successful outcome; previous studies were conducted in vitro (*4*). Miossec et al. (*1*) reported a series of 9 cases; all 9 patients had a medical history of stem cell or solid organ transplantation, and 8 died. The only survivor was a patient considered immunocompetent with no identified underlying conditions. A fatal *Microascus* sp. lung infection was previously published in a lung transplant recipient (*6*). Here, we report 2 lung transplant recipients infected with *M. cirrosus*, a ubiquitous mold isolated from soil and moist indoor environments (*7*). The third case we report was a young immunocompetent adult with no underlying conditions infected with *M. melanosporus*; his exposure by falling in a rice silo and sustaining serious injuries may explain the onset of opportunistic infection. 

*Microascus* spp. and *Scopulariopsis* (*8*) exhibit a multidrug-resistant phenotype (*9*). Skóra et al. reported antifungal susceptibility results of several *Microascus* species and confirmed high resistance to ciclopirox, 5-fluorocytosine, amphotericin B, and azoles. However, among echinocandin, lower minimum effective concentrations for caspofungin were reported (*10*). The highest in vitro activity was observed with terbinafin (*10*); synergistic activity was observed against some *Scopulariopsis* strains (*9*). Wiederhold et al. reported promising activity of olorofim on *Scopulariopsis* spp. and *Microascus* spp. fungi (*5*), but no synergistic in vitro activity was reported between olorofim and terbinafine against *Microascus* spp.

Appendix Additional information about cases of refractory microascus bronchopulmonary infection, France.
