# Supplementary material for: Refractory Microascus Bronchopulmonary Infection Treated with Olorofim, France
Source: Emerg Infect Dis. 2023 Nov;29(11):2401–3. doi: 10.3201/eid2911.230984 (PMC10617333; doi:10.3201/eid2911.230984)
Supplement: Appendix — Additional information about cases of refractory microascus bronchopulmonary infection, France. [file 23-0984-Techapp-s1.pdf]

Article DOI: https://doi.org/10.3201/eid2911.230984

EID cannot ensure accessibility for supplementary materials supplied by authors. Readers who have difficulty accessing supplementary content should contact the authors for assistance.

Refractory *Microascus* Bronchopulmonary Infection Treated with Olorofim, France

Appendix

Appendix Table. Antifungal susceptibility testing of *Microascus* spp. strains from 3 cases of refractory microascus bronchopulmonary infection, France*

Case	Strain	AmB	VCZ	PSZ	ITZ	ISZ	TBF	CAS	MIC	OLO
1	*M. melanosporus*	8	8	8	16	4	0,25	4	4	0.25
2	*M. cirrosus*	8	8	16	16	2	2	8	8	0.06
3	*M. cirrosus*	8	2	2	16	2	2	8	8	0.03

*AmB, amphotericine B; CAS, caspofungin; ISZ, isavuconazole; ITZ, itraconazole; MIC, micafungin; OLO, olorofim; PSZ, posaconazole; TBF, terbinafin; VCZ, voriconazole.

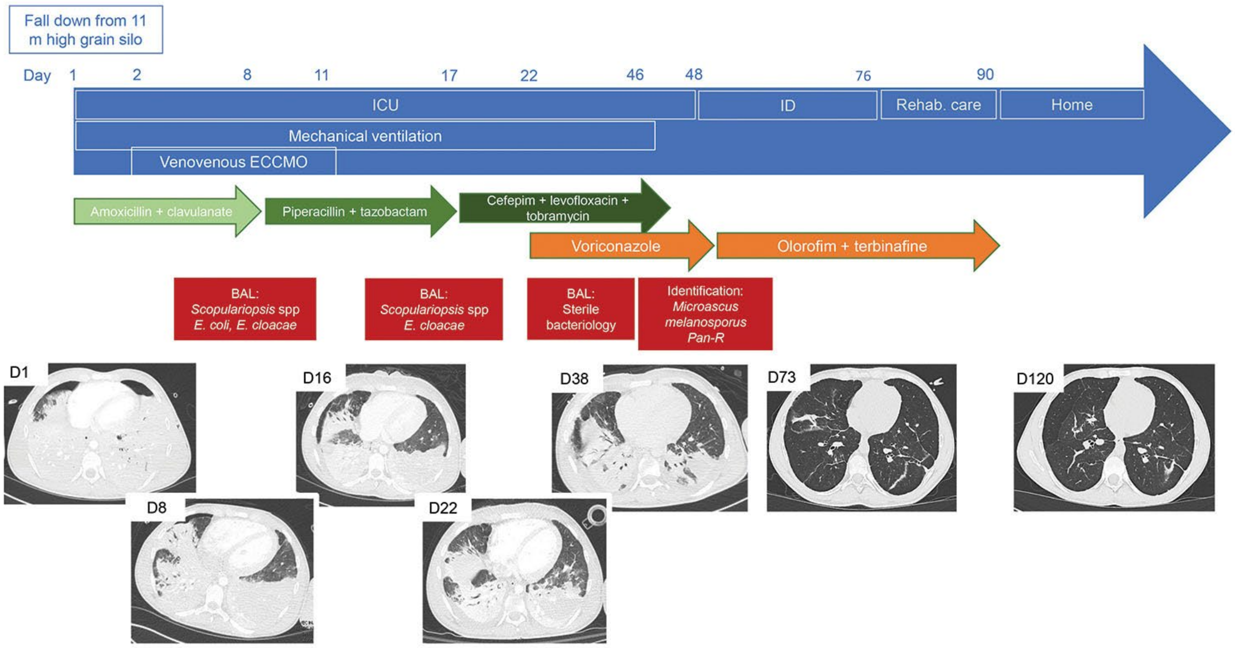

Appendix Figure 1. A) Summary of a case of refractory microascus bronchopulmonary infection in a patient 17 years of age. Day 1 (D1) is the admission in ICU just after the patient fell from a height of 11 m. Green arrows represent the successive antimicrobial therapies for bacterial respiratory infection. Orange arrows are the different antifungal therapies for fungal respiratory infection. Red arrows are the results of

bronchoalveolar lavage fluid cultures. B) Chest computed tomography scans performed during follow-up or at worsening of clinical status. ICU, intensive care unit; ECCMO, extracorporeal continuous membrane oxygenation; ID, infectious disease department; rehab care: rehabilitation care unit; home: day when patient was discharged to his home.

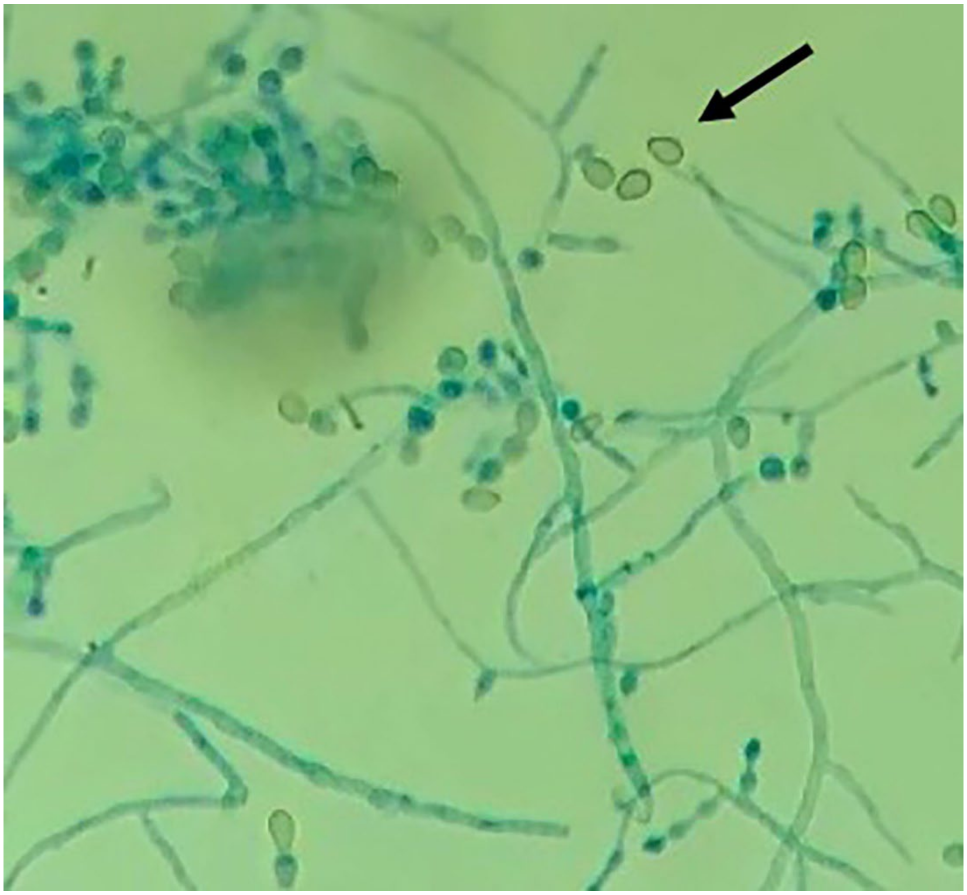

Appendix Figure 2. Microscopy examination (original magnification ×40) of strain of *Microascus melanosporus* after lactophenol cotton-blue stain showing vegetative hyphae, annellides, and truncate conidia.

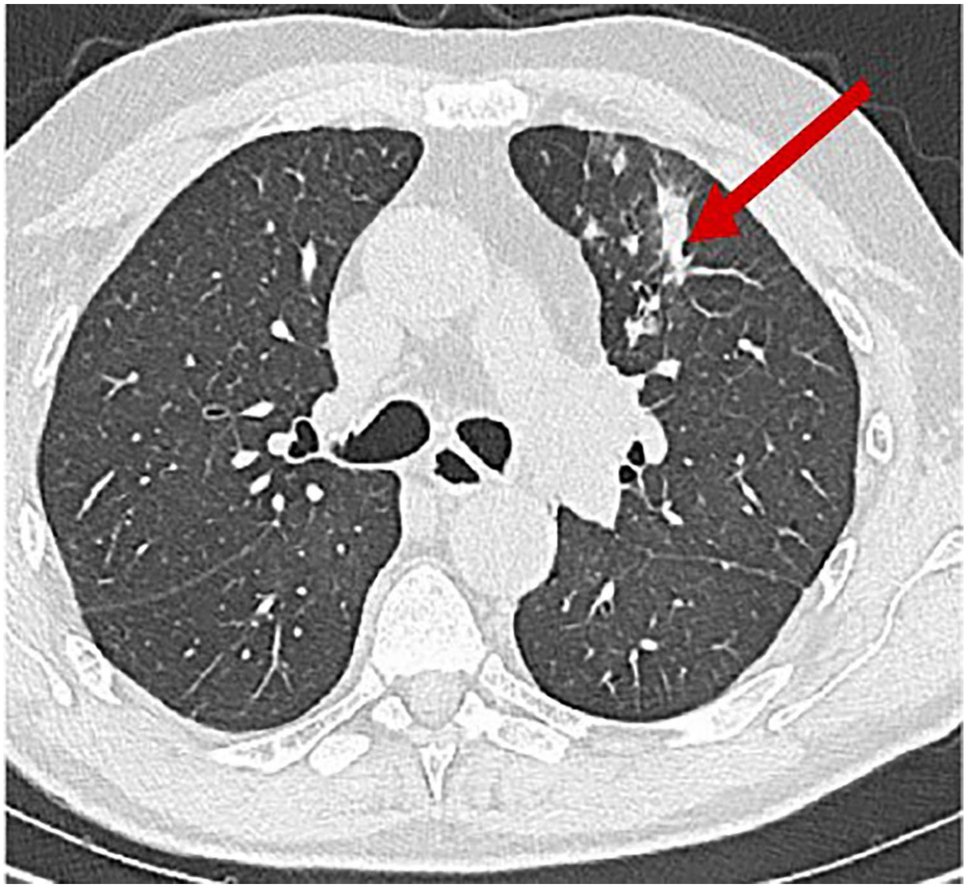

Appendix Figure 3. Thoracic computed tomography scan of patient with refractory microascus bronchopulmonary infection. Arrow indicates new-onset alveolar condensation in left upper lobe.

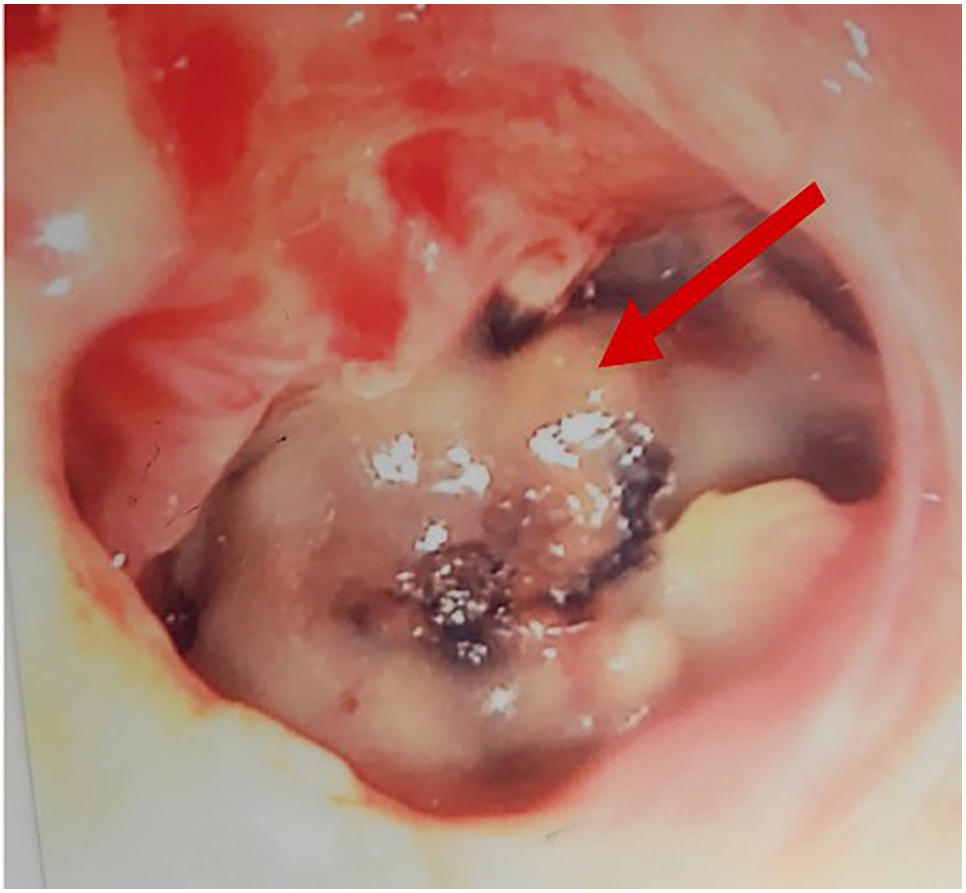

Appendix Figure 4. Macroscopic observation of endobronchial *Microascus cirrosus* lesion in case-patient 3 before olorofim treatment. Arrow indicates the lesion.
